# Physical activity and structured exercise patterns in perimenopausal women: a cross-sectional study

**DOI:** 10.3389/fgwh.2026.1933210

**Published:** 2026-10-07

**Authors:** Adriana Duaso-Iriarte, Eduardo Piedrafita, Alejandro Sarría, Héctor Gutiérrez

**Affiliations:** Faculty of Health Sciences, Universidad San Jorge, Villanueva de Gállego, Zaragoza, Spain

**Keywords:** endurance training, exercise behavior, exercise supervision, healthy aging, perimenopause, physical activity, strength training, women's health

## Abstract

Perimenopause is a critical midlife transition within women's aging, with implications for cardiometabolic, musculoskeletal, functional, and psychosocial health. Although physical activity and structured exercise are relevant components of healthy aging, evidence remains limited regarding how perimenopausal women organize their real-world exercise behavior, including exercise modalities and supervision. This cross-sectional study used a primarily descriptive approach to characterize physical activity habits and exercise patterns in perimenopausal women. Specifically, weekly physical activity level and intensity, exercise modalities, reported training time, and supervision status were examined, together with secondary exploratory comparisons across physical activity categories and according to selected sociodemographic and reproductive variables. An anonymous online questionnaire was completed by 121 women; 57 met the eligibility criteria, and 51 were included in the final analyses after exclusion of responses that did not allow calculation of total metabolic equivalent minutes per week (MET·min/week). Weekly physical activity was assessed using the International Physical Activity Questionnaire—Short Form. Mean total physical activity was 2258.1 ± 1447.3 MET·min/week. Moderate and high physical activity levels predominated, accounting for 43.1% and 39.2% of participants, respectively, whereas 17.6% were classified as having low physical activity. Endurance training (66.7%) and strength training (62.7%) were the most frequently reported exercise modalities, while sport-based activities were less common (35.3%). Endurance and strength training were reported more frequently in the higher physical activity categories. No statistically significant differences in total physical activity were identified according to the sociodemographic and reproductive variables examined, although a secondary exploratory comparison showed greater reported weekly strength training time among women aged 50 years or older than among younger participants. Participants reporting fully or partially supervised exercise showed descriptively higher total physical activity and more frequent participation in the exercise modalities examined, although the inferential comparisons of total physical activity and reported training time were not statistically significant. These findings provide a descriptive account of heterogeneous physical activity and exercise patterns in the sample examined. Given the achieved sample size and exploratory nature of the subgroup comparisons, the observed differences require confirmation in larger and more comprehensively characterized samples.

## 1 Introduction

Over recent decades, scientific interest in physical activity (PA) and exercise in women has increased substantially, contributing to a growing body of evidence on their relevance for health across the female lifespan. Nevertheless, women remain underrepresented in sports, exercise, and PA research, which has contributed to persistent gaps in sex-specific evidence and to an incomplete understanding of how female-specific life stages should be considered in research design and interpretation (1, 2). This evidence gap is particularly relevant for women in midlife, for whom reproductive aging, symptom burden, changing social roles, and emerging chronic disease risk may interact with PA behavior and long-term health trajectories (3).

Perimenopause represents a critical midlife transition within women's aging. It is characterized by progressive reproductive and endocrine changes, typically beginning with menstrual cycle variability and extending until one year after the final menstrual period. Beyond this biological transition, perimenopause may affect physical, emotional, mental, and social well-being, and is commonly accompanied by vasomotor symptoms, sleep disturbances, mood changes, and metabolic alterations that may influence health-related behaviors, including PA (4, 5).

The menopausal transition is also relevant from a healthy aging perspective because it is associated with changes in body composition, fat distribution, cardiometabolic risk, bone health, sleep, mood, and perceived quality of life (4–7). Given that natural menopause commonly occurs between 45 and 55 years of age, and that the menopausal transition precedes a prolonged post-reproductive stage for many women, behaviors adopted during midlife may have implications for functional preservation, chronic disease prevention, and long-term well-being (5, 7).

In this context, PA and structured exercise are widely recognized as relevant non-pharmacological components of healthy aging in women. Regular PA has been associated with reduced mortality in postmenopausal women, while evidence across the menopausal transition supports its relevance for body composition, fat distribution, cardiometabolic health, and musculoskeletal preservation (7–9). In particular, leisure-time PA has been associated with slower bone mineral density loss during the menopausal transition, supporting the importance of maintaining active lifestyles during midlife and beyond (9).

Recent overviews of reviews and systematic reviews suggest that PA, exercise, and broader lifestyle-oriented approaches may improve some menopause-related outcomes, including symptom burden, psychological well-being, and quality of life. However, findings remain heterogeneous across interventions and outcomes, and the variability in symptoms, health status, preferences, sociodemographic characteristics, and individual circumstances during the menopausal transition makes it unlikely that a single exercise or lifestyle approach will be optimal for all women (10, 11). This uncertainty highlights the value of characterizing how perimenopausal women actually engage in PA and structure their exercise practice in everyday settings.

Although reproductive and endocrine changes provide an important biological context for perimenopause, PA and exercise behavior during this stage are likely shaped by a broader set of interacting factors, including symptom burden, perceived health, motivation, time availability, social support, previous exercise experience, and access to appropriate guidance. Consequently, perimenopausal women should not be approached as a homogeneous group in terms of exercise engagement. Characterizing their real-world PA patterns, preferred exercise modalities, and degree of supervision may help identify behavioral profiles that are relevant to healthy aging and to future stage-sensitive recommendations (3, 4, 12).

Despite the recognized health relevance of PA, insufficient activity remains a major public health concern. Recent global estimates indicate that approximately one third of adults do not meet recommended PA levels, with insufficient PA being more prevalent among women than men (13). This issue is particularly relevant in midlife women, in whom physical inactivity may compound the accumulation of cardiometabolic, musculoskeletal, functional, and psychosocial health risks during the menopausal transition (3, 7, 12, 14).

However, while the physiological and clinical relevance of PA and exercise during the menopausal transition has received increasing attention, less is known about how perimenopausal women actually organize their PA and exercise behavior in real-world contexts. This includes the types of exercise they perform, the extent to which strength and endurance training are incorporated into their routines, and whether exercise is performed with professional supervision (3, 12, 15).

This gap is relevant because total PA volume alone may not fully capture the health-related meaning of exercise behavior during perimenopause. Women with similar weekly activity levels may differ substantially in the type, structure, and context of their practice, including whether they engage in strength training, endurance-oriented exercise, sport-based activities, or supervised sessions. This distinction is particularly important because exercise modalities such as strength training may have specific relevance for musculoskeletal and cardiometabolic health in women, while their uptake may be influenced by barriers related to knowledge, confidence, accessibility, supervision, and routine (7, 12, 15, 16).

On this basis, an exploratory characterization of PA and exercise patterns in perimenopausal women may provide useful population-specific evidence for understanding behavioral heterogeneity during this transition. Rather than assuming a uniform activity profile, this approach allows differences in PA level, exercise modality, supervision, and sociodemographic or reproductive context to be examined.

Therefore, the primary aim of the present study was to describe PA levels and structured exercise patterns in perimenopausal women, including weekly PA volume and intensity, exercise modalities, reported weekly training time, and supervision status. A secondary exploratory aim was to compare exercise variables across International Physical Activity Questionnaire—Short Form (IPAQ-SF) PA categories and to examine differences in PA and exercise variables according to selected sociodemographic and reproductive characteristics. Healthy aging was used as a conceptual framework for considering the relevance of PA during midlife and not as a clinical classification of the participants.

## 2 Materials and methods

### 2.1 Study design

A cross-sectional study with a primarily descriptive and secondary exploratory analytical approach was conducted to characterize PA levels and structured exercise patterns in perimenopausal women. The descriptive analyses addressed weekly PA volume and level, exercise modalities, reported weekly training time, and supervision status. Secondary exploratory comparisons examined exercise variables across IPAQ-SF PA categories and PA and exercise variables according to selected sociodemographic and reproductive characteristics, including age, marital status, presence of children, place of residence, educational level, and employment status.

The study was conducted in accordance with the ethical principles of the Declaration of Helsinki and was approved by the Ethics Committee of Universidad San Jorge (ref. 106/2026). Data collection was conducted over a predefined four-week period using an anonymous, self-administered online questionnaire.

The study was reported in accordance with the Strengthening the Reporting of Observational Studies in Epidemiology (STROBE) guidelines for cross-sectional studies (17).

### 2.2 Participants

The target population consisted of perimenopausal women. Eligibility was assessed according to the following inclusion criteria: (i) women aged between 45 and 55 years, inclusive; and (ii) a positive response to a screening item asking whether the participant met at least one of the following conditions: having received a diagnosis of perimenopause within the previous three months; experiencing irregular menstrual cycles with episodes of amenorrhea lasting more than 60 days but less than 11 months, not attributable to pregnancy or medication use; or experiencing variability of ≥7 days in cycle length over the previous 10 menstrual cycles, not attributable to medication use. The menstrual-cycle criteria were based on the Stages of Reproductive Aging Workshop +10 (STRAW+10) framework (18). The recent diagnosis of perimenopause was self-reported.

Exclusion criteria included: (i) current use of medication affecting the menstrual cycle, (ii) pregnancy, and (iii) current use of hormone replacement therapy, because exogenous hormonal treatment may modify bleeding patterns and thereby complicate the interpretation of menstrual-cycle-based eligibility criteria.

A total of 121 women completed the questionnaire during the predefined four-week data collection period. After applying the eligibility criteria, 64 responses were excluded because participants did not meet one or more inclusion criteria and/or met one or more exclusion criteria, resulting in 57 eligible perimenopausal women. Of these, 6 were excluded from the final analyses because their responses to IPAQ-SF did not allow the calculation of total MET·min/week, which was a core study variable. Therefore, the final analytical sample consisted of 51 women recruited through convenience sampling. The participant recruitment, eligibility assessment, and analytical flow are summarized in Figure 1.

**Figure 1:**
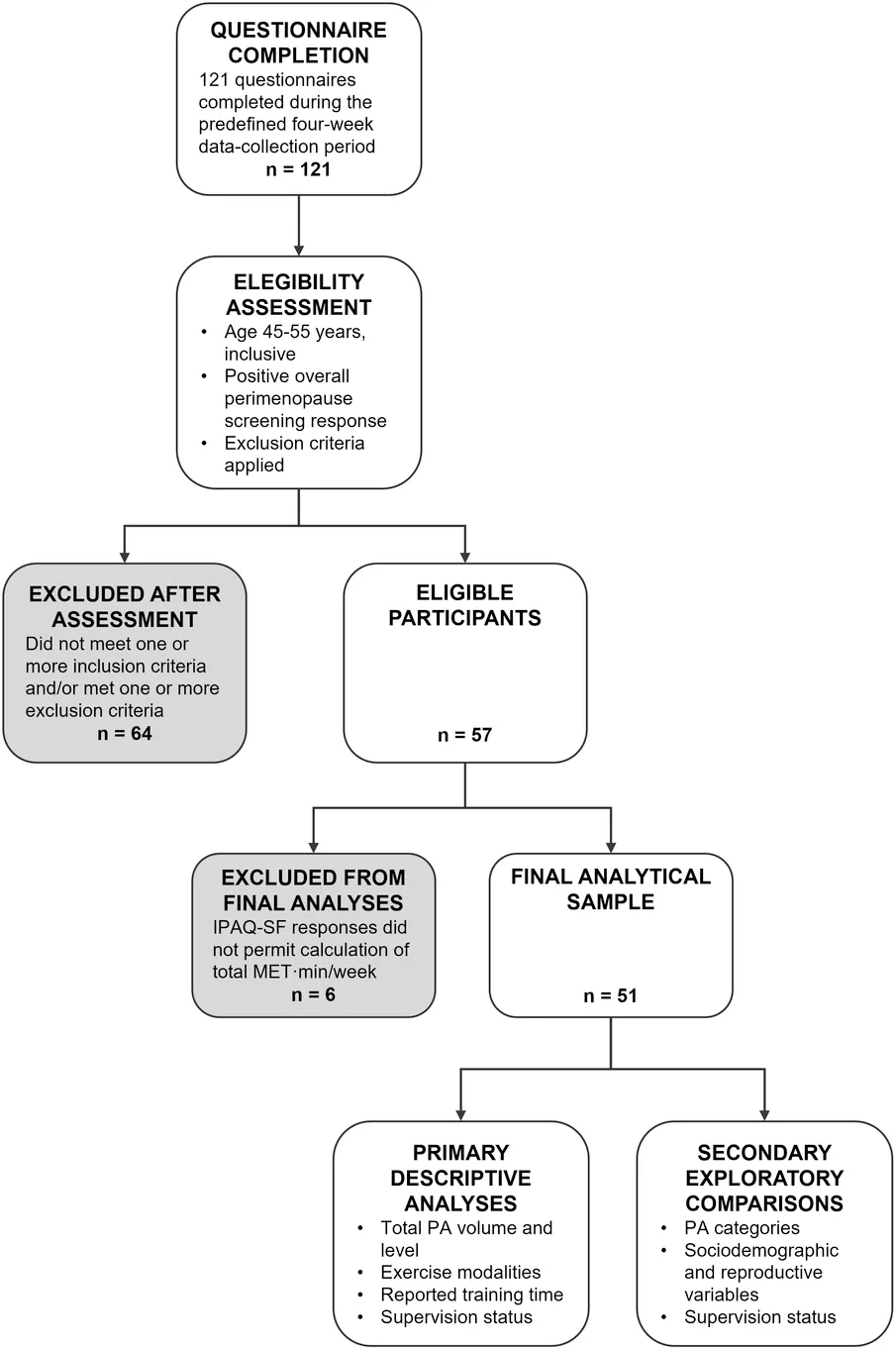
Methodological flowchart of participant recruitment, eligibility assessment, final analytical sample, and analytical approach. Eligibility for perimenopause was based on a single overall yes/no response indicating that at least one of the prespecified conditions was met. Six eligible participants were excluded from the final analyses because their IPAQ-SF responses did not allow calculation of total MET·min/week. IPAQ-SF, International Physical Activity Questionnaire—Short Form; MET, metabolic equivalent; PA, physical activity.

### 2.3 Sample size and analytical scope

An *a priori* sample size calculation was conducted during the study planning stage using G*Power software, version 3.1 (19). A one-way fixed-effects omnibus ANOVA comparing three independent PA categories was specified, with an alpha level of 0.05, statistical power of 1−*β* = 0.90, and a moderate effect size of *f* = 0.25 according to Cohen's convention (20). The resulting minimum estimated sample size was 207 participants.

The planned sample size was not achieved within the predefined four-week recruitment period. During this period, 121 questionnaires were completed, of which 57 met the eligibility criteria and 51 provided sufficient IPAQ-SF data for calculation of total MET·min/week and inclusion in the final analyses. Consequently, the study was treated as primarily descriptive, and all subgroup comparisons were considered secondary and exploratory rather than confirmatory.

### 2.4 Recruitment and data collection

Recruitment and data collection followed the predefined study period. Participants were recruited using a non-probability convenience sampling strategy through a multi-channel approach, including dissemination through social media and voluntary collaboration with healthcare and fitness-related centers.

The questionnaire was hosted on Microsoft Forms (Microsoft 365, Universidad San Jorge institutional license) and remained open for four weeks. Participation was voluntary, anonymous, and without financial compensation.

Written informed consent was waived because the study was conducted through an anonymous online questionnaire and no personally identifiable data were collected. Participants were informed about the purpose of the study, the voluntary nature of participation, and data confidentiality before completing the questionnaire. Completion of the questionnaire was considered as agreement to participate. No personally identifiable data were collected, and all responses were recorded and analyzed anonymously in accordance with applicable data protection regulations.

### 2.5 Instruments and variables

Weekly PA levels were assessed using the IPAQ-SF, a seven-item self-administered instrument that records the frequency (days/week) and duration (minutes/day) of walking, moderate-intensity activity, and vigorous-intensity activity performed over the previous seven days, as well as sitting time. The questionnaire was originally validated across 12 countries by Craig et al. (21), and has also been specifically validated in climacteric women (22).

Total PA was expressed in metabolic equivalent minutes per week (MET·min/week), calculated by multiplying minutes per day by days per week and by MET values according to IPAQ scoring guidelines (21): 3.3 METs for walking, 4.0 METs for moderate activity, and 8.0 METs for vigorous activity. Cases with incomplete or non-interpretable IPAQ-SF responses that prevented the calculation of total MET·min/week were excluded from the analytical dataset.

Participants were classified into three PA categories:

- High: ≥3 days of vigorous activity accumulating ≥1,500 MET·min/week, or ≥7 days of combined activity accumulating ≥3,000 MET·min/week.
- Moderate: ≥3 days of vigorous activity ≥20 min/day, or ≥5 days of moderate activity and/or walking ≥30 min/day, or ≥5 days of combined activity accumulating ≥600 MET·min/week.
- Low: not meeting the above criteria.

In addition to IPAQ-SF, the questionnaire included self-reported variables such as type of exercise performed (strength, endurance, and sport-based activities) and weekly duration, professional supervision of exercise practice, and sociodemographic variables including age, municipality size and category, employment status, educational level, marital status, presence of children, and age of children. The questionnaire was designed to characterize PA, exercise modalities, reported training time, supervision status, and selected sociodemographic and reproductive variables; it was not intended to provide a comprehensive clinical or functional health assessment.

For analytical purposes, selected sociodemographic variables were grouped into predefined categories. Age was categorized as <50 years and ≥50 years. Municipality size was classified as <200,000 or ≥200,000 inhabitants. Marital status was grouped as with partner or without partner; the latter category included women who were single, separated, divorced, or widowed. Employment status was categorized as with employment contract or without employment contract; the former included part-time and full-time contracted work, whereas the latter included unemployment and unpaid work. Educational level was grouped according to the highest educational attainment into three categories, defined within the Spanish educational system: level 1 (secondary/post-secondary non-university education), level 2 (university degree), and level 3 (postgraduate education and doctorate). Presence of children was categorized as yes/no. Among participants with children, additional descriptive information was collected on offspring age. Specifically, 44 participants reported having children, with a total of 51 children across these participants; of these, 17 were aged <12 years and 34 were aged ≥12 years. Because some participants had children in both age categories, offspring age was treated as descriptive family-context information rather than as a mutually exclusive participant-level grouping variable.

### 2.6 Statistical analysis

Statistical analyses were performed using IBM SPSS Statistics for Windows, version 30.0 (IBM Corp., Armonk, NY, USA). The distribution of continuous variables was assessed using the Shapiro–Wilk test, and homogeneity of variances was evaluated using Levene's test.

Descriptive statistics were calculated for all variables. Continuous variables are presented as mean ± standard deviation (SD), whereas dichotomous variables are reported as absolute frequencies and percentages. For proportions, 95% confidence intervals (CI) were estimated using the Wilson method when appropriate. Percentages for frequencies and “yes” responses were calculated over the total number of participants in the corresponding group.

Inferential analyses were performed according to the number of categories and the distributional assumptions of each variable. For comparisons involving two groups, independent-samples *t*-tests were used when normality and homogeneity assumptions were met, with Welch correction applied when appropriate; otherwise, the Mann–Whitney U test was used. For comparisons involving three groups, one-way ANOVA was used for normally distributed variables, whereas the Kruskal–Wallis test was applied when parametric assumptions were not met.

Effect sizes were calculated to quantify the magnitude of between-group differences. For comparisons involving two groups, Hedges’ *g* was used. For one-way ANOVA models, eta squared (*η*^2^) was calculated. For non-parametric analyses, Hedges’ *g* was estimated from the observed group means and SD to facilitate interpretation of the magnitude of between-group differences, whereas an approximate *η*^2^ coefficient was calculated for Kruskal–Wallis analyses using the formula *η*^2^ = (H−k + 1)/(n−k), where H is the Kruskal–Wallis statistic, k is the number of groups, and n is the total sample size.

Statistical significance was set at *p* ≤ 0.05. Given the achieved sample size and the small and unequal subgroup sizes, all inferential comparisons were considered exploratory. Effect sizes were reported to describe the magnitude of the observed between-group differences. No adjustment for multiple comparisons was applied because the subgroup analyses were exploratory; accordingly, individual *p* values were interpreted cautiously alongside effect sizes and descriptive patterns, without treating non-significant findings as evidence of equivalence or absence of a difference, or statistically significant findings as confirmatory evidence.

## 3 Results

### 3.1 Sample characteristics

Of the 121 women who completed the questionnaire during the predefined four-week recruitment period, 57 met the eligibility criteria for inclusion in the study. Six of these women were excluded from the final analyses because their responses to the IPAQ-SF did not allow the calculation of total MET·min/week, which was a core study variable. Therefore, the final analytical sample consisted of 51 perimenopausal women. The sociodemographic characteristics of the analytical sample are summarized in Table 1.

**Table 1:** Sociodemographic characteristics of the analytical sample (*n* = 51).

Variable	Category	n (%)
Age	<50 years	28 (54.9%)
	≥50 years	23 (45.1%)
Marital status	With partner	41 (80.4%)
	Without partner	10 (19.6%)
Children	Yes	44 (86.3%)
	No	7 (13.7%)
Educational level	Level 1	18 (35.3%)
	Level 2	18 (35.3%)
	Level 3	15 (29.4%)
Employment status	With employment contract	46 (90.2%)
	Without employment contract	5 (9.8%)
Municipality size	≥200,000 inhabitants	26 (51.0%)
	<200,000 inhabitants	25 (49.0%)

Percentages were calculated based on the analytical sample with valid IPAQ-SF data allowing the calculation of total MET·min/week (*n* = 51). Participant flow was as follows: 121 completed questionnaires, 57 eligible women after application of the inclusion and exclusion criteria, and 51 women included in the final analyses. Educational level was grouped according to the highest educational attainment within the Spanish educational system as follows: level 1, secondary/post-secondary non-university education; level 2, university degree; and level 3, postgraduate education and doctorate.

Overall, the sample showed a relatively balanced age distribution within the target perimenopausal range, with 54.9% of participants aged under 50 years and 45.1% aged 50 years or older. Most participants had a partner (80.4%), had children (86.3%), and reported having an employment contract (90.2%). Educational level was relatively evenly distributed across categories, with 35.3% of women classified in level 1, 35.3% in level 2, and 29.4% in level 3.

Regarding place of residence, participants were almost equally distributed between municipalities with 200,000 or more inhabitants (51.0%) and those with fewer than 200,000 inhabitants (49.0%). Taken together, these data indicate variability across the main sociodemographic variables considered in the study, providing descriptive context for the planned exploratory subgroup analyses.

### 3.2 Physical activity levels and exercise patterns

PA levels and exercise patterns in the analytical sample are presented in Table 2. Mean total PA was 2258.1 ± 1447.3 MET·min/week. According to the IPAQ-SF classification, the largest proportion of participants was categorized as having a moderate level of PA (43.1%), followed by a high level (39.2%), whereas a smaller proportion was classified as having a low level of PA (17.6%).

**Table 2:** Physical activity levels and exercise patterns in the analytical sample (*n* = 51).

Domain	Category	Measure	Result
Total physical activity	Total weekly PA	mean ± SD (MET·min/week)	2258.1 ± 1447.3
IPAQ-SF physical activity level	Low	n (%)	9 (17.6%)
	Moderate		22 (43.1%)
	High		20 (39.2%)
Exercise modality	Endurance training	n (%)	34 (66.7%)
	Strength training		32 (62.7%)
	Sport-based activities		18 (35.3%)

Continuous variables are presented as mean ± standard deviation (SD), and categorical variables as n (%). Percentages were calculated based on the analytical sample (*n* = 51) and may not total 100% because of rounding. Physical activity level was classified according to IPAQ-SF criteria as low, moderate, or high. Exercise modalities were self-reported, and categories were not mutually exclusive. PA, physical activity; IPAQ-SF, International Physical Activity Questionnaire—Short Form.

Regarding exercise modalities, endurance training was the most frequently reported type of exercise, practiced by 66.7% of participants. Strength training was also commonly reported, with 62.7% of women indicating engagement in this modality, while sport-based activities were less prevalent (35.3%). Within the analytical sample, endurance and strength training were reported more frequently than sport-based activities.

### 3.3 Exploratory comparisons according to physical activity level

Comparisons of activity-related continuous variables according to IPAQ-SF physical activity level are presented in Table 3, and the distribution of exercise modalities across PA categories is summarized in Table 4.

**Table 3:** Activity-related continuous variables according to IPAQ-SF physical activity level in the analytical sample (*n* = 51).

Outcome	Low (*n* = 9)	Moderate (*n* = 22)	High (*n* = 20)	*p* value	Effect size
Total physical activity (MET·min/week)	715.1 ± 453.3	1484.5 ± 666.9	3559.6 ± 1160.5	<0.001	*η*^2^ = 0.63
Reported weekly endurance training (min/week)	125.0 ± 90.0	70.0 ± 49.2	116.5 ± 99.5	0.35	η^2^ = 0.08
Reported weekly strength training (min/week)	86.7 ± 35.1	75.4 ± 45.8	151.3 ± 100.6	0.05	η^2^ = 0.19
Reported weekly sport-based activities (min/week)	87.5 ± 46.0	80.0 ± 31.5	121.7 ± 75.9	0.35	η^2^ = 0.12

Continuous variables are presented as mean ± standard deviation (SD). *p* values correspond to overall between-group comparisons according to the tests selected based on distributional assumptions: Kruskal–Wallis tests for total physical activity and endurance training minutes, and one-way ANOVA for strength training and sport-based activity minutes. Statistical significance was defined as *p* ≤ 0.05. Effect sizes are presented as eta squared (η^2^). IPAQ-SF: International Physical Activity Questionnaire—Short Form.

**Table 4:** Exercise modalities according to IPAQ-SF physical activity level in the analytical sample (*n* = 51).

Exercise modality	Low (*n* = 9)	Moderate (*n* = 22)	High (*n* = 20)
Endurance training	4 (44.4%)	11 (50.0%)	19 (95.0%)
Strength training	3 (33.3%)	12 (54.5%)	17 (85.0%)
Sport-based activities	2 (22.2%)	8 (36.4%)	8 (40.0%)

Values are presented as *n* (%), with percentages calculated within each IPAQ-SF physical activity level category. Exercise modalities were self-reported, categories were not mutually exclusive, and modality frequencies were not formally compared across physical activity categories. IPAQ-SF: International Physical Activity Questionnaire—Short Form.

As expected from the IPAQ-SF classification criteria, significant overall differences were observed in total PA, with mean values increasing progressively across groups from low (715.1 ± 453.3 MET·min/week) to moderate (1484.5 ± 666.9 MET·min/week) and high PA levels (3559.6 ± 1160.5 MET·min/week; *p* < 0.001, *η*^2^ = 0.63).

Regarding reported weekly training time, no significant overall differences were found across PA levels for endurance training minutes (*p* = 0.35, *η*^2^ = 0.08) or sport-based activity minutes (*p* = 0.35, *η*^2^ = 0.12). In the exploratory comparison of reported weekly strength training time, the overall difference reached the predefined statistical threshold (*p* = 0.05, *η*^2^ = 0.19), with the highest mean observed in the high PA group (151.3 ± 100.6 min/week), compared with the moderate (75.4 ± 45.8 min/week) and low groups (86.7 ± 35.1 min/week).

The descriptive distribution of exercise modalities across PA categories is presented in Table 4. Endurance training was reported by 44.4% of women in the low PA group, 50.0% in the moderate group, and 95.0% in the high group. Similarly, strength training was reported by 33.3%, 54.5%, and 85.0% of participants in the low, moderate, and high PA groups, respectively. Sport-based activities were reported by 22.2% of women in the low group, 36.4% in the moderate group, and 40.0% in the high group. These modality frequencies were not formally compared.

Taken together, these descriptive findings show that endurance and strength training were reported more frequently in the higher IPAQ-SF categories. Exploratory comparisons of reported weekly training time identified an overall difference for strength training, whereas no statistically significant overall differences were identified for endurance training or sport-based activities.

### 3.4 Exploratory comparisons according to sociodemographic and reproductive variables

Secondary exploratory comparisons according to sociodemographic and reproductive variables are presented in Table 5. Overall, no statistically significant differences in total PA were observed according to municipality size, employment status, educational level, marital status, presence of children, or age group (all *p* > 0.05).

**Table 5:** Total physical activity and weekly strength training according to sociodemographic and reproductive variables in the analytical sample (*n* = 51).

Grouping variable	Category	N (%)	Total physical activity (MET·min/week)	*p* value	Effect size	Weekly strength training (min/week)	*p* value	Effect size
Municipality size	≥200,000 inhabitants	26 (51.0%)	2251.9 ± 1368.4	0.98	*g* = 0.01	110.4 ± 57.7	0.75	*g* = 0.11
	<200,000 inhabitants	25 (49.0%)	2265.1 ± 1566.7			120.0 ± 104.8		
Employment status	With contract	46 (90.2%)	2324.0 ± 1481.2	0.39	*g* = 0.50	117.4 ± 86.8	0.81	*g* = 0.31
	Without contract	5 (9.8%)	1582.5 ± 895.1			90.0 ± 84.9		
Educational level	Level 1	18 (35.3%)	2463.4 ± 1722.5	0.80	η^2^ = 0.01	116.3 ± 68.9	0.11	η^2^ = 0.13
	Level 2	18 (35.3%)	2176.3 ± 1248.2			151.8 ± 107.1		
	Level 3	15 (29.4%)	2134.5 ± 1410.3			82.1 ± 63.1		
Marital status	With partner	41 (80.4%)	2137.8 ± 1429.0	0.27	*g* = 0.37	116.2 ± 89.5	0.94	*g* = 0.03
	Without partner	10 (19.6%)	2679.1 ± 1507.6			113.3 ± 73.9		
Children	Yes	44 (86.3%)	2190.3 ± 1455.4	0.39	*g* = 0.29	109.8 ± 90.4	0.21	*g* = 0.34
	No	7 (13.7%)	2625.9 ± 1453.0			140.0 ± 62.0		
Age group	<50 years	28 (54.9%)	1966.2 ± 1153.5	0.13	*g* = 0.45	97.1 ± 98.4	0.02	*g* = 0.47
	≥50 years	23 (45.1%)	2622.9 ± 1708.1			138.2 ± 63.0		

Continuous variables are presented as mean ± standard deviation (SD). *p* values correspond to between-group comparisons according to the tests selected based on distributional assumptions in the analytical dataset. Hedges’ *g* was used as the effect size for two-group comparisons, and eta squared (η^2^) for educational level, which included three categories. Statistical significance was defined as *p* ≤ 0.05. Educational level categories were defined according to the Spanish educational system, as described in the Methods section.

Mean total PA was very similar according to municipality size (2251.9 ± 1368.4 vs. 2265.1 ± 1566.7 MET·min/week; *p* = 0.98, *g* = 0.01). Likewise, no clear differences were found according to educational level, with mean values ranging from 2134.5 ± 1410.3 to 2463.4 ± 1722.5 MET·min/week across categories (*p* = 0.80, *η*^2^ = 0.01).

Mean total PA was numerically higher among women aged 50 years or older than among those aged under 50 years (2622.9 ± 1708.1 vs. 1966.2 ± 1153.5 MET·min/week), but this exploratory difference was not statistically significant (*p* = 0.13, *g* = 0.45). Mean total PA was also numerically higher among women without a partner than among those with a partner (2679.1 ± 1507.6 vs. 2137.8 ± 1429.0 MET·min/week), but this exploratory difference was not statistically significant (*p* = 0.27, *g* = 0.37). Similarly, mean total PA was numerically higher among women without children than among those with children (2625.9 ± 1453.0 vs. 2190.3 ± 1455.4 MET·min/week), but this exploratory difference was not statistically significant (*p* = 0.39, *g* = 0.29).

Most exercise-related comparisons across sociodemographic and reproductive variables were also non-significant. In the exploratory comparison according to age group, reported weekly strength training time was higher among participants aged 50 years or older than among younger participants (138.2 ± 63.0 vs. 97.1 ± 98.4 min/week; *p* = 0.02, *g* = 0.47). No significant differences were found in weekly endurance training minutes or sport-based activity minutes across the remaining sociodemographic variables.

Taken together, these exploratory comparisons did not identify statistically significant differences in total PA according to the sociodemographic and reproductive variables examined. The only statistically significant subgroup difference concerned reported weekly strength training minutes according to age group; however, given the achieved sample size and multiple exploratory comparisons, this finding should be interpreted cautiously.

### 3.5 Supervised exercise

Supervision status in the analytical sample and its distribution across PA categories are summarized in Table 6. Of the 51 women included in the analytical sample, 21 reported that their exercise was supervised, 9 reported that some sessions were supervised, 11 reported no supervision, and 10 did not respond to this questionnaire item. Thus, 30 participants were classified as performing fully or partially supervised exercise, 11 as exercising without supervision, and 10 as missing for this item.

**Table 6:** Exercise supervision status in the analytical sample and according to IPAQ-SF physical activity level (*n* = 51).

Supervision status	Total sample (*n* = 51)	Low PA (*n* = 9)	Moderate PA (*n* = 22)	High PA (*n* = 20)
Yes	21 (41.2%)	2 (22.2%)	9 (40.9%)	10 (50.0%)
Some sessions	9 (17.6%)	1 (11.1%)	2 (9.1%)	6 (30.0%)
No	11 (21.6%)	3 (33.3%)	6 (27.3%)	2 (10.0%)
No response	10 (19.6%)	3 (33.3%)	5 (22.7%)	2 (10.0%)
Fully/partially supervised	30 (58.8%)	3 (33.3%)	11 (50.0%)	16 (80.0%)

Values are presented as *n* (%), with percentages calculated within each physical activity category. Of the 51 women included in the analytical sample, 10 did not respond to the supervision item. For descriptive purposes, the category “Fully/partially supervised” was created by combining the responses “Yes” and “Some sessions”. IPAQ-SF, International Physical Activity Questionnaire—Short Form; PA, physical activity.

Descriptively, the proportion of women reporting fully or partially supervised exercise increased across PA categories. Among women classified as having a low level of PA, 3 of 9 reported supervised or partially supervised exercise, compared with 11 of 22 in the moderate group and 16 of 20 in the high PA group.

Comparisons between participants with and without supervised exercise among those with available data are presented in Table 7. Mean total PA was numerically higher among women reporting fully or partially supervised exercise than among those exercising without supervision (2633.4 ± 1407.4 vs. 1973.9 ± 1671.6 MET·min/week), but this exploratory difference was not statistically significant (*p* = 0.24, *g* = 0.44).

**Table 7:** Total physical activity and exercise characteristics according to supervision status among participants with available data (*n* = 41).

Domain	Measure	Fully/partially supervised (*n* = 30)	No supervision (*n* = 11)	*p* value	Effect size
Supervision status	n (%)	30 (73.2%)	11 (26.8%)	Not applicable	
Total physical activity	mean ± SD (MET·min/week)	2633.4 ± 1407.4	1973.9 ± 1671.6	0.24	*g* = 0.44
Endurance training	n (%)	24 (80.0%)	6 (54.5%)	Not formally tested	
Strength training		24 (80.0%)	4 (36.4%)		
Sport-based activities		18 (60.0%)	0 (0.0%)		
Endurance training time	mean ± SD (min/week)	105.9 ± 89.5	103.0 ± 101.8	0.83	*g* = 0.03
Strength training time		127.8 ± 93.1	78.8 ± 37.5	0.36	*g* = 0.54
Sport-based activity time		100.5 ± 59.1	Not estimable		

Continuous variables are presented as mean ± standard deviation (SD), and categorical variables as *n* (%). Percentages for exercise modalities were calculated within each supervision category. Supervision data were available for 41 of the 51 participants, as 10 participants did not respond to this questionnaire item. *p* values correspond to between-group comparisons according to the tests selected based on distributional assumptions in the analytical dataset. Hedges’ *g* was used as the effect size for two-group comparisons. Statistical significance was defined as *p* ≤ 0.05. “Not applicable” indicates that no inferential comparison was performed for the row describing group composition itself. Exercise modality frequency rows are shown descriptively and were not formally tested in this table. “Not estimable” indicates that inferential comparison was not possible because no participant in the unsupervised group reported that modality.

Descriptively, supervised participants more frequently reported endurance training (24/30, 80.0% vs. 6/11, 54.5%), strength training (24/30, 80.0% vs. 4/11, 36.4%), and sport-based activities (18/30, 60.0% vs. 0/11, 0.0%) than those exercising without supervision.

Regarding weekly exercise time, no statistically significant differences were observed between groups for endurance training minutes (*p* = 0.83, *g* = 0.03) or strength training minutes (*p* = 0.36, *g* = 0.54). Mean weekly endurance training time was 105.9 ± 89.5 min/week in the supervised group and 103.0 ± 101.8 min/week in the unsupervised group, whereas mean weekly strength training time was 127.8 ± 93.1 and 78.8 ± 37.5 min/week, respectively.

Among participants with available supervision data, women reporting fully or partially supervised exercise showed descriptively higher total PA and more frequent participation in the three exercise modalities examined. These patterns were descriptive, and the inferential comparisons of total PA and reported weekly training time did not reach statistical significance.

## 4 Discussion

The present study primarily provides a descriptive characterization of PA levels, exercise modalities, reported weekly training time, and supervision status in a convenience sample of perimenopausal women. Secondary exploratory comparisons examined exercise variables across IPAQ-SF PA categories and PA and exercise variables according to selected sociodemographic and reproductive characteristics. Moderate and high PA levels predominated, and endurance and strength training were the most frequently reported exercise modalities. The exploratory comparisons also showed descriptive differences in exercise modality distribution across PA categories and supervision groups. However, given the achieved sample size and small and unequal subgroups, these comparisons should not be interpreted as confirmatory evidence of between-group differences.

Healthy aging provides the conceptual context for considering the potential relevance of PA and structured exercise during the menopausal transition. However, healthy aging was not assessed as an outcome or used to classify the participants’ health status. The available data therefore characterize PA and exercise patterns in perimenopausal women but do not establish that the sample represented a clinically healthy or healthy-aging population.

These findings should be interpreted within the broader context of perimenopause as a midlife transition characterized by reproductive and endocrine changes and considerable variability in symptoms and health-related experiences (4, 5, 7, 12). The present study contributes sample-specific descriptive information on reported PA volume, exercise modalities, and supervision status. However, symptoms, health status, exercise motivations, and individual support needs were not assessed, and the observed variation in exercise behavior should not be interpreted as evidence of specific health or intervention needs.

### 4.1 Physical activity levels and exercise modalities

Regarding weekly PA level, moderate and high IPAQ-SF categories predominated in the present sample, whereas fewer participants were classified in the low PA category. Endurance and strength training were also reported more frequently in the higher PA categories. These findings describe heterogeneity in PA volume and reported exercise modality within the sample but do not establish the participants’ motivations, the regularity or duration of their previous exercise practice, or whether their exercise was undertaken for health-related purposes.

This distribution differs somewhat from reports in other populations of menopausal or midlife women, in which insufficient PA or only moderate adherence to healthy lifestyle behaviors remains common. For example, El Hajj et al. reported a high prevalence of low PA among women living in the Mediterranean region, whereas Alnjadat et al. found that many menopausal women did not exercise weekly and that overall adherence to diet and PA recommendations was moderate (14, 23). These differences may be partly explained by sampling strategy, recruitment channels, cultural context, and the possibility that women interested in exercise or menopause-related self-management were more likely to participate in the present survey. Therefore, although the relatively favorable PA distribution observed in our sample is informative, it should not be interpreted as representative of all perimenopausal women.

From a healthy aging perspective, PA during the menopausal transition is relevant because previous longitudinal research has linked PA with physical performance and the preservation of bone mineral density during the menopausal transition and postmenopause (9, 24). However, the present study did not assess physical performance, bone health, cardiometabolic outcomes, or other health indicators. The IPAQ-SF categories and MET·min/week values should therefore be interpreted as descriptive measures of reported PA rather than as indicators of broader health-related behavioral organization or health status.

With regard to exercise modalities, endurance and strength training were the most frequently reported forms of exercise in the present sample, practiced by 66.7% and 62.7% of participants, respectively. Both modalities were reported more frequently in the higher IPAQ-SF PA categories. In the exploratory comparisons of reported weekly training time, the overall difference reached the predefined statistical threshold for strength training (*p* = 0.05), with the highest mean observed in the high PA category, whereas no statistically significant overall difference was identified for endurance training time (*p* = 0.35). Previous evidence supports the potential relevance of resistance-oriented exercise for strength, bone-related outcomes, and metabolic or cardiovascular parameters during the menopausal transition, although the optimal type and dose of strength training remain insufficiently established (25). Previous research has also identified knowledge, confidence, accessibility, supervision, time efficiency, exercise preferences, perceived relevance, and program structure as factors that may influence participation in strength and endurance exercise among women (15, 16). However, these factors were not assessed in the present study, and the observed frequency and duration patterns cannot be attributed to specific motivations, previous experience, intended health outcomes, or healthy-aging behaviors.

Sport-based activities were less frequently reported than endurance and strength training, with 35.3% of participants reporting this modality. Their descriptive frequency increased from 22.2% in the low PA category to 36.4% in the moderate category and 40.0% in the high category, although these frequencies were not formally compared. Reported weekly sport-based activity time also did not differ statistically across IPAQ-SF PA categories (*p* = 0.35). The present data do not indicate whether the lower frequency of sport-based activities reflected accessibility, preference, confidence, previous experience, scheduling requirements, perceived performance demands, or other contextual factors. Consequently, the findings should not be interpreted as evidence that sport-based activities are less beneficial, suitable, or desired by perimenopausal women (3, 12, 15). Future quantitative and qualitative studies should examine the factors underlying exercise-modality selection and participation during the menopausal transition.

### 4.2 Exercise supervision and clinical implications

Among participants with available supervision data, those reporting fully or partially supervised exercise had numerically higher mean total PA and more frequently reported endurance training, strength training, and sport-based activities than those reporting no supervision. However, the inferential comparisons of total PA and reported weekly training time were not statistically significant, and the exercise-modality frequencies were not formally compared. These findings should therefore be considered descriptive. The study did not assess why participants sought supervision or whether supervision was related to health conditions, medical advice, previous experience, technical support, motivation, or personal preference.

Previous literature indicates that motivation, self-efficacy, social support, time constraints, perceived competence, previous exercise experience, and access to guidance may influence exercise participation in midlife women (3, 12, 15, 26). Supervision may also provide technical guidance for modalities such as strength training (16). These considerations provide context for future research but do not explain the descriptive findings of the present study, because these potential mechanisms were not measured. Nevertheless, recording supervision status alongside PA volume and exercise modality may contribute to a more detailed descriptive characterization of exercise practice.

Previous resistance-training research suggests that supervised approaches may improve some training outcomes compared with unsupervised or self-guided formats, particularly for strength-related measures, although this evidence is not specific to perimenopausal women and may vary according to population, intervention characteristics, and outcomes assessed (27, 28). In the present sample, reported weekly strength training time was numerically higher in the supervised group, but the exploratory between-group comparison was not statistically significant. Strength training was also reported more frequently in the supervised group, although these frequencies were not formally compared. The present findings therefore do not establish whether supervision influenced exercise participation or whether women with particular exercise patterns were more likely to seek supervision. Prospective and intervention studies are needed to determine whether supervised approaches influence training participation, adherence, safety, or health-related outcomes during perimenopause.

From a clinical and health-promotion perspective, the descriptive findings of the present study suggest that PA volume may be considered alongside exercise modality, reported weekly training time, and supervision context when characterizing exercise behavior during the menopausal transition. Total MET·min/week provides an estimate of overall PA volume but does not describe whether endurance, strength, or sport-based activities are included in an individual's routine or whether exercise is performed with professional supervision. Recording these complementary dimensions could provide healthcare and exercise professionals with additional descriptive information and may inform subsequent individualized assessment of symptoms, health conditions, previous exercise experience, barriers, preferences, and access to appropriate guidance. However, the present study did not examine whether this broader characterization improves clinical assessment or decision-making, and it did not assess clinical outcomes, menopausal symptoms, functional status, exercise motivations, or the effectiveness of supervised exercise. Accordingly, the findings do not support a specific clinical prescription or establish the superiority of any exercise modality or supervision model. Any exercise recommendations during the menopausal transition should remain individualized and based on appropriate clinical assessment, current evidence-based guidance, participant preferences, accessibility, and safety considerations.

### 4.3 Sociodemographic and reproductive characteristics

In the exploratory comparisons according to sociodemographic and reproductive characteristics, no statistically significant differences in total PA were identified according to municipality size, employment status, educational level, marital status, presence of children, or age group. Previous studies have identified age, educational level, family-related characteristics, motivation, self-efficacy, and social support as potential correlates of PA or adherence to health-related behaviors in midlife and menopausal women (23, 29, 30). However, the absence of statistically significant differences in the present study should not be interpreted as evidence that the characteristics examined are unrelated to PA or as contradicting previous literature, given the achieved sample size, small and unequal subgroups, convenience sampling strategy, and differences in the populations, outcomes, and analytical approaches examined. Furthermore, the study did not assess clinical, behavioral, motivational, or contextual factors such as motivation, self-efficacy, social support, preferences, barriers, or sustainability that might have contributed to the observed PA and exercise patterns.

The only statistically significant result in this set of exploratory comparisons was greater reported weekly strength training time among participants aged 50 years or older than among younger participants. This finding should be interpreted cautiously because age was dichotomized, the subgroup analysis was exploratory, multiple comparisons were performed, and the study was not adequately powered for confirmatory inference. The present data do not indicate why reported strength training time differed between the age groups and cannot establish whether the difference was related to menopausal stage, health status, awareness of musculoskeletal health, previous exercise experience, motivation, or other factors. Although previous literature supports the relevance of strength training during midlife and has identified several factors that may influence participation (15, 16), the age-related difference observed here requires confirmation in larger and more comprehensively characterized samples.

### 4.4 Strengths and limitations

Several aspects of the study may nevertheless contribute useful descriptive information. First, the study focused specifically on women meeting the prespecified perimenopausal eligibility criteria rather than on a broader, undifferentiated midlife sample. Second, the questionnaire captured several complementary dimensions of PA and exercise behavior, including total reported PA volume, IPAQ-SF categories, exercise modalities, reported weekly training time, and supervision status. Third, use of the IPAQ-SF provides consistency with previous PA research, and the instrument has been specifically evaluated in climacteric women (21, 22). Within the limitations of the sampling strategy and collected variables, the study therefore provides exploratory descriptive information on how PA and exercise were reported in this sample.

Several limitations should be considered when interpreting these findings. First, although an *a priori* sample size calculation estimated that 207 participants would be required for the originally planned three-group comparison under the specified assumptions, the final analytical sample comprised 51 participants. The study therefore did not achieve the statistical power originally planned for confirmatory subgroup analyses, particularly for detecting small-to-moderate differences and for comparisons involving small and unequal groups. Non-significant findings should consequently not be interpreted as evidence of equivalence or as confirmation that no between-group difference exists, and all subgroup comparisons should be regarded as exploratory and hypothesis-generating. Second, the cross-sectional design does not allow causal inference or temporal ordering between PA patterns, exercise modalities, supervision status, and the characteristics examined (31).

Third, convenience sampling may limit transferability, because recruitment through social media and healthcare or fitness-related centers may have favored participation among women with greater interest in exercise or menopause-related self-management. The predominance of moderate and high PA levels further suggests that the sample may represent a comparatively active subgroup of perimenopausal women rather than the broader population. Fourth, the questionnaire was not designed to provide a comprehensive clinical or functional characterization of the participants. Body mass index, smoking status, chronic diseases, general health and functional status, medication use beyond the prespecified exclusion criterion, menopausal symptom burden, previous exercise history or duration, reasons for exercising, reasons for selecting particular exercise modalities, and reasons for seeking supervision were not assessed. Perimenopausal eligibility was based on a single overall response indicating that at least one of the prespecified conditions was met; therefore, the specific eligibility pathway and overlap between criteria could not be determined retrospectively. These factors limit the clinical and reproductive-stage characterization of the sample and may influence the interpretation of the observed between-participant and between-group differences. Furthermore, although the exclusion of current hormone replacement therapy users helped preserve the interpretability of the menstrual-cycle-based eligibility criteria, it limits the applicability of the findings to the broader population of women undergoing the menopausal transition. Accordingly, healthy aging should be understood as the conceptual framework of the study rather than as a measured characteristic of the sample.

Fifth, PA and exercise characteristics were self-reported and may therefore be affected by recall bias, social desirability, and misclassification; future studies may benefit from combining self-report instruments with device-based PA assessment (32). Finally, missing supervision data reduced the sample available for supervised versus unsupervised comparisons. Future research should include larger and more diverse samples, objective PA measures, quantitative or qualitative assessments of health status, menopausal symptoms, exercise history, motivations, barriers, preferences, and reasons for supervision, as well as separate recording of each reproductive-stage eligibility criterion and additional information on the source and basis of any reported diagnosis.

In conclusion, this study provides a descriptive account of PA levels and structured exercise patterns in a convenience sample of perimenopausal women. Moderate and high PA levels predominated, and endurance and strength training were the most frequently reported exercise modalities. In the secondary exploratory comparisons, a statistically significant overall difference in reported weekly strength training time was identified across IPAQ-SF PA categories, and reported weekly strength training time was significantly higher among participants aged 50 years or older than among younger participants. No statistically significant differences in total PA were identified according to the sociodemographic and reproductive characteristics examined or according to supervision status, and no statistically significant differences in reported weekly endurance or strength training time were identified according to supervision status. Differences in exercise-modality frequencies across IPAQ-SF PA categories and supervision groups were descriptive and were not formally tested. Given the achieved sample size, multiple exploratory comparisons, and small and unequal subgroups, the statistically significant and descriptive findings require confirmation in larger and more comprehensively characterized samples. The results support the value of describing exercise modality, reported training time, and supervision context alongside total PA volume, while future studies should examine the health and functional implications of these patterns.

## Data Availability

The datasets generated and analyzed during the current study are not publicly available due to privacy and ethical considerations related to participant data. Anonymized data may be made available by the corresponding author upon reasonable request.
